# Editorial: Artificial intelligence and Internet of Things for smart agriculture

**DOI:** 10.3389/fpls.2024.1494279

**Published:** 2024-10-02

**Authors:** Shanwen Zhang, Chuanlei Zhang, Ce Yang, Bin Liu

**Affiliations:** ^1^ Information Engineering College, Xijing University, Xi’an, China; ^2^ College of Artificial Intelligence, Tianjin University of Science and Technology, Tianjin, China; ^3^ Department of Bioproducts and Biosystems Engineering, University of Minnesota, Saint Paul, MN, United States; ^4^ School of Information Engineering, Northwest A&F University, Xi’an, China

**Keywords:** smart agriculture, Agricultural Internet of Things (AIoT), Agricultural Digital Twin (ADT), Agricultural UAV(AUAV), Agricultural Remote Sensing (ARS)

## 1 Introduction

In recent years, in the field of smart agriculture, many advanced technologies and data-driven approaches have been widely applied to crop yield estimation, soil and water conservation, pest and disease detection and severity evaluation, species classification and farmland ridge segmentation, and have achieved remarkable results. This Research Topic highlights several issues that still need further research and discussion in smart agriculture, such as Agricultural UAV (AUAV), Agricultural Remote Sensing (ARS), Agricultural Internet of Things (AIoT), Agricultural Artificial Intelligence (AAI), Agriculture Digital Twin (ADT), Deep Learning and their combinations.

## 2 AUAV and ARS

AUAV and ARS can capture field surface information, provide a wide range of agricultural monitoring, and help farmers and agricultural managers make better decisions. In recent years, combining AUAV, ARS and AI have been widely applied to real-time monitoring and analysis of crop growth status, crop health status and growth stage, and assessment of the impact of natural disasters, such as droughts, floods and storms, and help farmers understand the field situation and develop responses and reduce risks in time. Garofalo et al. highlighted the potential of combining AUAV, ARS and AI in precision agriculture, with the goal of effectively monitoring physiological parameters. By analyzing AUAV and ARS data, farmers can optimize fertilization, irrigation, and pest control strategies to improve crop yield and quality, reduce waste and promote sustainable agricultural development. The application of AUAV, ARS and AI technologies is constantly developing, and combined with big data and deep learning, will provide more powerful support for agricultural production in the future. Jafar et al. reviewed the methods, applications and limitations of AAI and AIoT in crop disease detection, provided detailed steps for crop disease prediction using AAI and AIoT, and discussed the application of machine learning and deep learning in crop pest detection, and pointed out the future research prospect of combining AAI with intelligent AUAV and AIoT for field disease detection and monitoring.

## 3 AIoT and mobile devices

AIoT and MDs aim to combine AIoT and MD with AI to provide solutions for smart agriculture. Various AIoT sensors are installed in the field to collect soil moisture, temperature, humidity, crop health, weather conditions and other data in real time, and transmit the collected data to the cloud platform to achieve optimal irrigation, fertilization, disease and pest control, and accurate resource management. MDs such as smartphone and AUAV are used to capture crop images in the field to realize real-time monitoring of farmland, including crop variety classification, crop disease severity estimation and pest detection (Pan et al.; Bedi et al.). The widespread application of AIoT and MDs is transforming the agricultural landscape, making it more efficient, sustainable, and able to meet the challenges of smart agriculture. Li et al. introduced a variable direction irrigation decision-making method of east-west ridge based on the AIoT management control system. This study provides a new irrigation decision-making way to improve the efficiency of crop production. Li et al. summarized the latest development of AAI and AIoT, and proposed an efficient deep learning architecture based on Mobile Vision Transformer (MobileViT) for real-time detection of crop disease. It is designed with high-accuracy and low-cost, making it suitable for deployment on MDs with limited resources. Qiu et al. proposed a method for measuring water content of millet by using AIoT differential capacitance sensor. It provides a reliable means for the accurate determination of crop water content, and provides a strong support for improving agricultural production efficiency and resource utilization.

## 4 Agricultural Digital Twin

Digital twins have been applied to various fields, including smart agriculture. ADT is one example of many digital farming innovations. It relies on the integration of state-of-the-art agricultural technologies, including big-data analytics, AAI, AIoT, ARS, AUAV, Information & Communication Technology, Geographic Information Systems (GIS). It helps farmers monitor crop growth, soil conditions and climate change in real time, optimize the use of water, fertilizers and pesticides, reduce resource waste and environmental impact, predict potential risks, such as pests and diseases, climate change, and take steps to reduce losses, create a comprehensive basis for data-driven policy decisions, and promote sustainable development of agriculture. Recently, ADT has promoted the application of new technologies such as AIoT, AAI and big-data analytics to smart agriculture, and become an important tool for modern agricultural management. The application of ADT will revolutionize agricultural production, improving overall efficiency and sustainability. Garske et al. reviewed “digital twin sustainable development”, “digital twin”, “digital twin agriculture”, “Data governance”, “Destination Earth”, “EU Data Strategy”, and “EU Data Governance Act”. By collecting and analyzing large amounts of data, ADT can provide scientific basis for agricultural management, facilitate data-driven decision-making processes, enable precision agriculture, drive sustainable development, reduce negative impacts on the environment, and implement the EU Green Deal-in line with internationally binding climate and environmental targets.

## 5 Conclusion

Smart agriculture integrates advanced agricultural technologies, including AUAV, ARS, AIoT, AAI, ADT and deep learning. It can provide farmers with tools and platforms for automated irrigation, fertilization, pest control, crop pest detection, growing environment monitoring, unmanned farm management and automatic crop harvesting, so as to improve efficiency and optimize agricultural production, minimizing environmental impact and resource use. At present, there are still several open research problems that need to be further studied and perfected. Due to the complexity and diversity of agricultural big-data, as well as its large scale and universal distribution, many challenges are presented in terms of network speed, computing storage, and operations management. This topic will introduce new achievements in the fields of AUAV, ARS, AIoT, AAI, ADT, deep learning and their integration and application in sustainable smart agriculture.

